# Spatiotemporal Analysis of Hydration Mechanism in Sodium Alginate Matrix Tablets

**DOI:** 10.3390/ma14030646

**Published:** 2021-01-30

**Authors:** Ewelina Juszczyk, Piotr Kulinowski, Ewelina Baran, Artur Birczyński, Dorota Majda, Encarna García-Montoya, Pilar Pérez-Lozano, Josep Maria Suñé-Negre, Władysław P. Węglarz, Przemysław Dorożyński

**Affiliations:** 1 Research and Development Center, Celon Pharma S.A., Marymoncka 15, 05-152 Kazuń Nowy, Poland; ewelina.juszczyk@celonpharma.com; 2 Institute of Technology, The Pedagogical University of Kraków, Podchorążych 2, 30-084 Kraków, Poland; ewelina.baran@up.krakow.pl (E.B.); artur.birczynski@up.krakow.pl (A.B.); 3 Faculty of Chemistry, Jagiellonian University, Gronostajowa 2, 30-387 Kraków, Poland; majda@chemia.uj.edu.pl; 4 Pharmaceutical Technology and Physico-Chemical Department, Universitat de Barcelona, Av. Joan XXIII, 27-31, 08028 Barcelona, Spain; encarnagarcia@ub.edu (E.G.-M.); perezlo@ub.edu (P.P.-L.); jmsune@ub.edu (J.M.S.-N.); 5 Department of Magnetic Resonance Imaging, Institute of Nuclear Physics Polish Academy of Sciences, Radzikowskiego 152, 31-342 Kraków, Poland; wladyslaw.weglarz@ifj.edu.pl; 6 Department of Drug Technology and Pharmaceutical Biotechnology, Medical University of Warsaw, Banacha 1, 02-097 Warszawa, Poland; przemyslaw.dorozynski@wum.edu.pl

**Keywords:** sodium alginate, mass transport, water–polymer interaction, spatial water distribution, compressed matrix tablets, hydrophilic polymeric matrices, magnetic resonance imaging (MRI), ultrashort echo time (UTE), multi-echo spin-echo (MSME) T_2_ relaxation time mapping, differential scanning calorimetry (DSC)

## Abstract

Methods of spatiotemporal characterization of nonequilibrated polymer based matrices are still immature and imperfect. The purpose of the study was to develop the methodology for the spatiotemporal characterization of water transport and properties in alginate tablets under hydration. The regions of low water content were spatially and temporally sampled using Karl Fisher and Differential Scanning Callorimetry (spatial distribution of freezing/nonfreezing water) with spatial resolution of 1 mm. In the regions of high water content, where sampling was infeasible due to gel/sol consistency, magnetic resonance imaging (MRI) enabled characterization with an order of magnitude higher spatial resolution. The minimally hydrated layer (MHL), infiltration layer (IL) and fully hydrated layer (FHL) were identified in the unilaterally hydrated matrices. The MHL gained water from the first hour of incubation (5–10% *w*/*w*) and at 4 h total water content was 29–39% with nonfreezing pool of 28–29%. The water content in the IL was 45–47% and at 4 h it reached ~50% with the nonfreezing pool of 28% and T_2_ relaxation time < 10 ms. The FHL consisted of gel and sol layer with water content of 85–86% with a nonfreezing pool of 11% at 4 h and T_2_ in the range 20–200 ms. Hybrid destructive/nondestructive analysis of alginate matrices under hydration was proposed. It allowed assessing the temporal changes of water distribution, its mobility and interaction with matrices in identified layers.

## 1. Introduction

Sodium alginate is a natural polymer used in various sectors including the food and pharmaceuticals industries. In particular, its pharmaceutical applications cover a broad spectrum of possibilities—it can be used as an encapsulation material (cell culture and transplantation), dental molds (impression material), a hemostatic and absorbent for wound dressings and a filler for the preparation of sustained-release tablets [1,2,3,4,5]. The understanding of phenomena occurring during mass transport within controlled drug delivery systems is a key factor for the rational design and development of such medicinal products. The hydration of controlled release matrix tablets based on hydrophilic polymers is a complex and dynamic process. It integrates the water transport (including capillary phenomena), dissolution and erosion of the polymer matrix, changing the rotational freedom of polymer chains under the influence of the solvent, polymer swelling and the formation of viscous solutions [6,7]. All these phenomena occur simultaneously in the tablet under hydration and influence its properties, which may have a great impact on its behavior inside gastrointestinal tract in humans.

Among the techniques which can be used to determine water content, Differential Scanning Calorimetry (DSC) and Karl Fischer titration (KF) are considered the “gold standard”. The latter is specific to water, which makes KF a reliable tool in studies concerning the transport/distribution of water. DSC is also favorably used to assess water–polymer interaction and other physicochemical properties in many papers focused on the polymers used in pharmaceutical technology. These methods are inherently destructive and are generally used to study homogeneous materials [8,9,10,11,12].

The multimodal study of water transport within the polymeric tablet as well as its interaction with the matrix is demanding due to the dynamic and nonequilibrated state of the material in experimental conditions along with susceptibility to disturbance of the observed phenomena. Starting from mid-1990s, magnetic resonance imaging (MRI) has been applied as a noninvasive technique for studies of oral drug delivery systems. From the very beginning the main problem of MRI measurements was “translating” the obtained qualitative or quantitative images into the physicochemical properties of the analyzed system (e.g., water content, viscosity, matrix–water interaction etc.). Several approaches to calibrate in situ MRI results using equilibrated samples have been presented [13,14]. The results of these studies cannot be fully satisfying due to obvious differences between equilibrated “molecularly static” calibrating sample properties and nonequilibrated “molecularly dynamic” experimental samples. Another aspect of the spatiotemporal imaging studies is that they are usually focused on the external “gel layer” (spatially resolved relaxometry or diffusometry). Studies concerning the full spatial cross-section range of the hydrated matrix including interfacial regions, i.e., between solid matrix and viscous “gel-like” regions, are still infrequent. The hypothesis of the work was that the spatiotemporal characteristics of nonequilibrated samples during hydration should lead to an understanding of the interplay of the processes occurring in real matrix systems, e.g., those commonly used in pharmacy. 

Therefore, the aim of the study was (1) to develop an integrated experimental methodology, which allows analyzing the physicochemical properties of polymer matrix tablets and matrix hydration mechanisms; (2) to obtain the spatial and temporal characteristics of the alginate compressed tablet under hydration in qualitative and quantitative manner.

## 2. Materials and Methods

### 2.1. Materials

Sodium alginate Protanal LF 240 D was supplied by FMC Biopolymers (Philadelphia, PA, USA): the intrinsic viscosity [η] = 4.27 × 10^2^ cm^3^/g, average molar mass 151,955 g/mol, degree of polymerization 767, M unit content: 65–70%, G unit content: 30–35% (M/G~2).

Aquametric Composite 5, methanol and methanol high purity grade were purchased from Panreac AppliChem (Barcelona, Spain). Hydranal Composite 5 and methanol were obtained from Honeywell Fluka (Charlotte, NC, USA). All other materials used in the study were of analytical grade.

### 2.2. Tablet Preparation and Hydration

Round, flat matrix tablets with a diameter of 12 mm, a thickness of 6 mm and a weight of 800 mg were prepared by direct compression of sodium alginate using a single punch tablet press EK0 (Korsch-Erweka, Berlin, Germany). The hardness of all tested tablets was 40–50 N.

**Figure 1:**
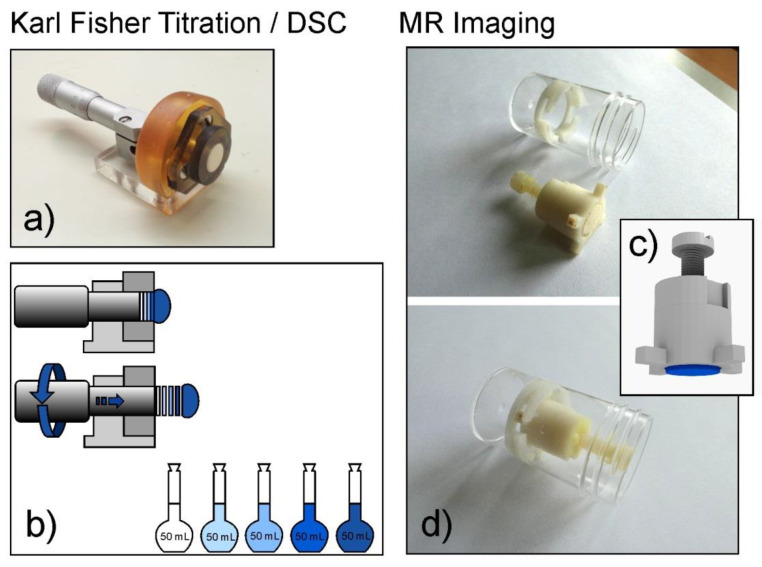
The equipment applied for sampling of the material in Karl Fischer (KF) and Differential Scanning Calorimetry (DSC) experiments and the corresponding holder for Magnetic Resonance Imaging (MRI) studies: (**a**) device for unilateral tablet hydration and localized sampling by tablet slicing; (**b**) a diagram depicting tablet slicing; (**c**) model of 3D printed nonmagnetic holder for MRI study; (**d**) 3D printed holder together with polycarbonate chamber.

For a spatially localized sampling of the hydrated tablet, a dedicated device was developed (Figure 1a). It consisted of a tablet holder, for unilateral hydration of the tablets, and a micrometric screw coupled with a piston, which allowed pushing up the tablet within the holder to the required position to enable localized sampling of the hydrated matrix material (Figure 1b).

For hydration, the tablet was tightly inserted into the device with the surface positioned at the level of the holder (see Figure 1a). Then it was submerged in 900 ml of distilled water at 37 °C. After 1, 2, 3 or 4 h the device was removed from the hydration medium. A swollen tablet material that stuck out of the holder was removed with a spatula and it was considered as a first “slice”. Next, the hydrated matrix remaining in the holder was cut into 1 mm thick slices (Figure 1b). About 50–100 mg of material was taken from the central part of each slice for Karl Fisher (KF) and differential scanning calorimetry (DSC) measurements.

### 2.3. Spatial Distribution of Water Content Within the Matrix Tablets Using Karl Fischer Titration

The samples were taken from the hydrated sodium alginate tablet at 1, 2, 3 and 4 h of hydration according to the procedure described in Section 2.2. They were accurately weighed, put into 50 mL flasks filled with methanol and put into a sonic bath for 15 min and subsequently left for 24 h for stabilization. The water content of the samples (*wc_tot(KF)_*) was determined using KF titration (890 Titrando with Touch Control and 803 Ti Stand; Metrohm, Herrisau, Switzerland) and corrected for blank measurements. All measurements were carried out in triplicate. The initial water content in unhydrated sodium alginate tablets was also determined.

### 2.4. Differential Scanning Calorimetry

The samples for DSC measurements were taken after 4 h of hydration according to the procedure described in Section 2.2. They were accurately weighed and closed hermetically in aluminum crucibles. Phase transition temperature and enthalpy of the transition of water were calibrated using distilled water as a standard.

Differential scanning calorimetry was carried out using the DSC 822^e^ (Mettler–Toledo, Greifensee, Switzerland) with a liquid nitrogen cooling station. The instrument was calibrated for temperature and enthalpy using zinc, n-octane and indium standards. The following thermal protocol was used for heating experiments: samples were frozen to −80 °C at 10 °C/min, and then they were gradually heated from −80 °C to 30 °C with a scanning rate of 2 °C/min. For each time point, two samples were measured. The total water content in the samples (*wc_tot(DSC)_*) was determined by the difference of the sample weight before and after its heating to 180 °C in unsealed crucibles. Mass of freezing water was determined based on the ratio of phase transition enthalpy *ΔH* to heat melting of ice *q_top_* = 332 mJ/mol [15]. Enthalpy of the process was equal to the area under the DSC curve. The freezing water content (*wc_f(DSC)_*) was calculated as the percentage of freezing water weight against the total sample weight. The content of nonfreezing water: *wc_nf(DSC)_* was calculated according to Equation (1) [11,12]:

*wcnf(DSC)* = *wctot(DSC)*−*wcf(DSC)* (1)

### 2.5. Magnetic Resonance Imaging and Image Analysis

The internal structure and water distribution within the matrix tablets were analyzed by MRI using a 9.4 T Bruker Biospin MRI scanner (Bruker, Ettlingen, Germany) operating at proton frequency of 400 MHz. The tablets were placed in the 3D printed nonmagnetic holders of similar construction to the device applied for KF and DSC studies (Figure 1c). The holders were then put into the 30 mL polycarbonate chambers (Figure 1d) filled with water. The chambers were placed in a thermostatic water bath at 37 °C ± 0.5 °C (LW 502M, AJL Electronic, Kraków, Poland). At 1, 2, 3 and 4 h of incubation, the chambers were withdrawn from the water bath and placed in the scanner. For the study purpose, two different imaging sequences were applied: multi-slice multi-echo (MSME) and ultrashort echo time (UTE).

The parameters of the MSME sequence were as follows: number of echoes—NE = 256, echo time—TE = 3.536 ms, repetition time—TR = 5 s, number of accumulations—NA = 1, slice thickness—1 mm, field of view—FOV = 28 × 28 mm^2^, matrix size of 256 × 256.

The following parameters were applied in UTE sequence: TE = 300 µs, TR = 30 ms, flip angle—15°, NA = 20, dummy scans, DS = 10; number of radial projections—804, number of sampling points for single projection—256, slice thickness—1 mm; field of view—FOV = 28 × 28 mm^2^, matrix size of 256 × 256.

UTE images and MSME stacks of images obtained at consecutive echo times were imported to Fiji distribution of ImageJ version 1.44 (National Institutes of Health, Bethesda, ME, USA, http://rsb.info.nih.gov/ij/) [16]. Each image or image stack was rotated to align holder edges in parallel with image borders. Next, a 14-pixel wide (1.53 mm) part of the image was selected and cropped—it covered whole matrix (including regions referred to as KF, DSC slices) and the surrounding hydration medium. The rows of the resulting images were averaged to obtain a profile along the axis of the tablet (1D image). For UTE data, the resulting intensity profile was plotted against the spatial position. For MSME data, pixel-by-pixel analysis of the 1D profile was performed to obtain 1D parametric image. For each profile pixel image intensity (*f*) vs. echo time (*TE*) was fitted using Levenberg–Marquard algorithm with exponential function Equation (2):

(2) $$f\left(t\right)=y_{0}+Ae^{-\frac{t}{T_{2}}}$$

where *y_0_* is constant level, *A*—amplitude, *T*_2_—effective T_2_ relaxation time. The calculations were made in OriginPro 2018 (OriginLab Corporation, MA, USA).

## 3. Results and Discussion

**Figure 2:**
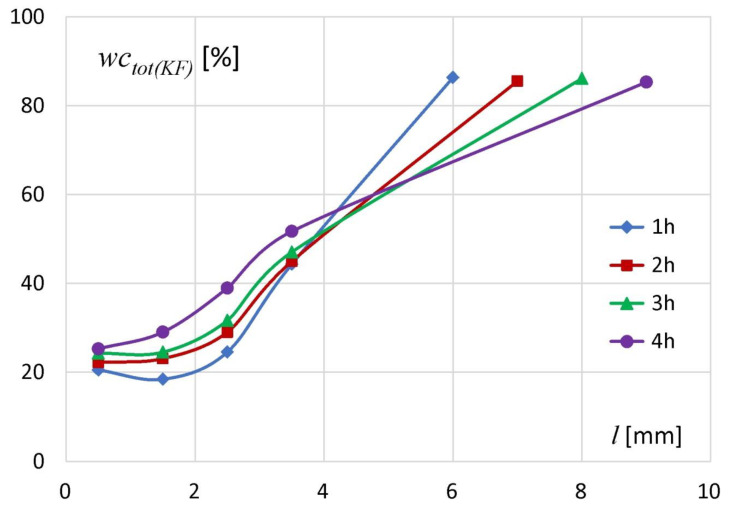
Changes of total water content (*wc_tot(KF_*_)_) in subsequent tablet slices at 1, 2, 3 and 4 h of hydration as measured using Karl Fischer method.

The results of the KF analysis are presented in Figure 2. Position *l* = 0 denotes the matrix bottom and spatial positions’ (*l*) increase towards the hydration medium (water). The initial water content of the unhydrated tablets containing sodium alginate determined by the KF (*wc_0(KF)_*) method was 15.2%. The values of total water content (*wc_tot(KF)_*) in all tablet slices were higher than the initial polymer water content, regardless of hydration time. It meant that the water penetrated into the deepest tablet regions even after the shortest measured hydration time (1 h). Along the axial section of the tablets, after the first hour of hydration the *wc_tot(KF)_* in particular slices was in the range of approximately 20–86%. After two hours of the experiment the increase of water content in particular slices was in the range 1–5%. The material from the slice located at *l* = 0–1 mm (tablet bottom) contained 22% of water. In the next slices the water content was 22.9% for *l* between 1 and 2 mm, 28.8% (for *l* between 2 and 3 mm) and 44.8% (for *l* between 3 and 4 mm). After three hours of the experiment, the changes in the amount of water in particular slices did not exceed 2.5% in comparison to the values obtained after 2 h of hydration. After four hours there was an increase in water content of approximately 1–7%. In subsequent slices starting from the tablet bottom (*l* from 0 to 4 mm) following water concentrations were measured: 25.1% (*l* between 0 and 1 mm), 28.9% (*l* between 1 and 2 mm), 38.9% (*l* between 2 and 3 mm) and 51.5% (*l* between 3 and 4 mm). KF studies showed that the uptake of water by the matrix was the highest in its external part close to the hydration medium. Water content in that region (*l* ~ 6–9 mm depending on incubation time) was approximately 85–86% and had similar values regardless of hydration time.

The DSC studies of the material taken from particular slices allowed for the characterization of the interaction between the water and the sodium alginate chains at 4 h of hydration. Endothermic peaks were observed during the heating run in the tablet for *l* between 0 and 9 mm. The peaks were the result of first-order phase transition of water present in the sample and it were called “freezing water”. In the slice located between *l* = 0 and 1 mm freezing water was not detected, as no peaks were registered according to experimental conditions used in the study [10,17].

**Figure 3:**
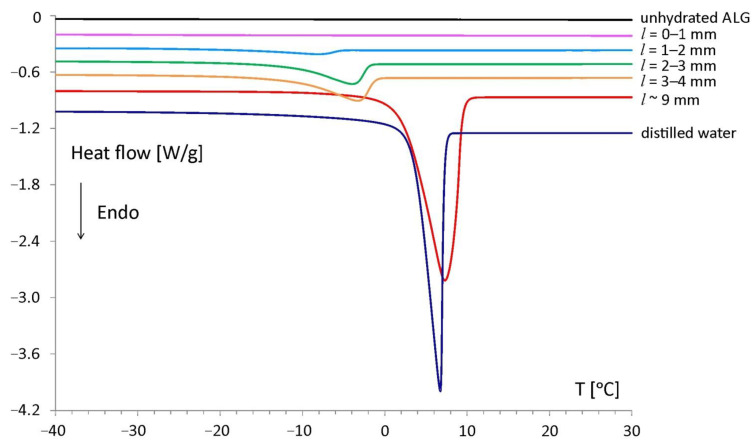
DSC heating curves of the alginate matrix slices obtained at 4 h of hydration, a thermogram of distilled water and unhydrated sodium alginate.

The DSC curve of the unhydrated polymer (Figure 3, black line) showed no phase transitions in the heating run. Therefore, the occurrence of DSC peaks in some slices of the hydrated sodium alginate tablet was associated with the phase transition of water penetrating the matrix during hydration. In spatial range (*l* = 0–10 mm) only one endothermic peak was observed. The normalized peak areas were increasing upon the distance from the tablet bottom, and it was associated with increasing freezing water content in the sample. The highest freezing water content was determined in the external part of the tablet, which had direct contact with the surrounding hydration medium (matrix region centered at *l* ~ 9 mm).

With decreasing water content, a gradual shift of onset temperatures towards lower temperatures was observed. The lowest temperature of the phase transition was observed at approximately −12 °C for the slice located between *l* = 1 and 2 mm. The shift of melting onsets in hydrated sodium alginate samples is also reported in other works [18,19]. The DSC characteristics of the hydrated sodium alginate matrix could be affected by the presence of sodium ions, which stay in the vicinity of the polymer chains [20,21]. The highest absolute value of phase transition heat was recorded in the external part of the tablet (*l* ~ 9 mm): −245 mJ/mg, and the lowest at a slice located between *l* = 1 and 2 mm was −13 mJ/mg. The onset of the water melting peak for *l* ~ 9 mm was recorded at +2 °C and was close to the melting temperature of pure water: +3 °C. This indicated the presence of free water in this matrix region.

**Figure 4:**
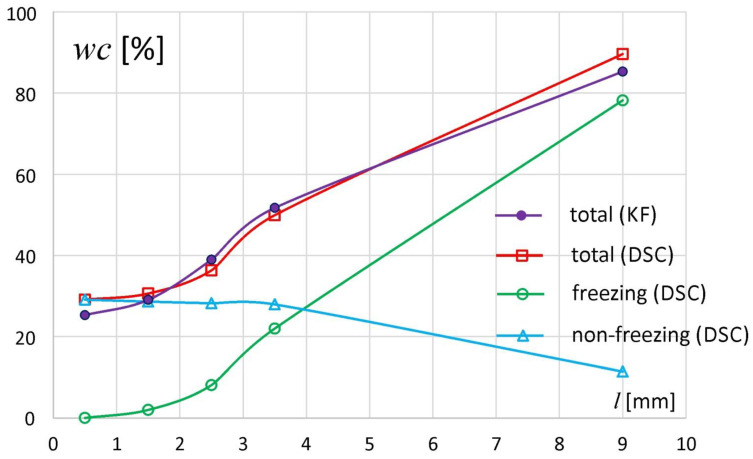
Freezing water content (blue), nonfreezing water (green) and total water (red) in different tablet regions (slices) of the sodium alginate matrix after four hours of hydration in water determined by the DSC method; and total water content (*wc_tot(KF)_*) determined by KF method (purple).

Both the unhydrated polymer and the samples taken from the tablet bottom (*l* between 0 and 1 mm) did not contain the freezing water fraction. However, the existence of water in these samples was confirmed by the difference in sample weight before and after its heating up to 180 °C according to the procedure described in the Section 2.4. The water molecules present in those samples were strongly restricted by the alginate chains and did not form a solid phase. This type of water was designated as nonfreezing water. Figure 4 presents the individual water fractions in the samples of all tablet slices—total, freezing and nonfreezing water content. For comparison, the total water content determined by KF method is also presented. The results of the measurements of the total content of water by the KF and DSC methods were convergent.

The distribution of water in the sodium alginate tablets indicated that the samples taken from the matrix regions (slices) above *l* = 1 mm contained both freezing and nonfreezing water in varying proportions. The highest content of freezing water was observed in the external part of the tablet and decreased gradually towards the tablet bottom. In the sample taken from the slice at *l* ~ 9 mm, *wc_f (DSC)_* = 79%, and the slice located between *l* = 1 and 2 mm contained only ca. 1% of freezing water. In contrast, the nonfreezing water content in the external tablet region was about 11%, while in the rest of the tablet it remained at a constant level of 28–29%. A constant level of nonfreezing water with total water content below 50% (as happened here) is also reported by Ping et al. during the hydration of polyvinyl alcohol [9].

**Figure 5:**
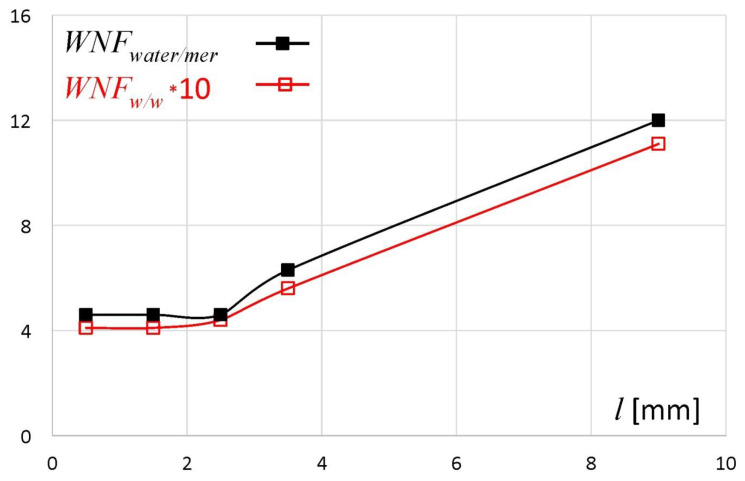
Nonfreezing water per one dry polymer unit expressed as *WNF_w/w_* and *WNF_water/mer_* (detailed description in the text) for subsequent sodium alginate matrix slices at 4 h of hydration.

The nonfreezing water fraction described above was expressed as a percentage of total sample weight (%). However, in the literature the ratio of nonfreezing water weight and dry polymer weight (*WNF_w/w_*) is commonly used. This is because the ratio *WNF_w/w_* better shows the interaction between water and polymer binding sites and its value does not directly depend on the total water content in the sample. In Figure 5 the fraction of nonfreezing water expressed as the ratio *WNF_w/w_* is presented. The same ratio was also recalculated as the number of water molecules per one dry polymer unit (*WNF_water/mer_*), where the molar mass of water is 18 g/mol and the molar mass of one unit of sodium alginate is 198 g/mol, and presented in the same graph [15,22]. The ratio *WNF_w/w_* remained at the level of ca. 0.4 in the slices/regions located between *l* = 0 and 2 mm, and then it ranged from 0.44 for *l* = 2–3 mm to 1.11 at *l* ~ 9 mm. Similar *WNF_w/w_* values were obtained for other water–sodium alginate systems [8,19,22]. An increase of *WNF_w/w_* above a certain total water content level was also observed for other polymer systems [23]. The values of *WNF_water/mer_* indicated that for one unit of dry polymer there were between 5 (for *l* = 0–1 mm) to approximately 12 (for *l* ~ 9 mm) of nonfreezing water molecules. Similar results are reported by Nakamura et al. [19]. Mazur et al. have estimated that in the first hydration shell of alginate anion in aqueous solutions there are six (± 2) water molecules with reduced rotational dynamics and five water molecules slowed down by the sodium ion (total: 11 ± 2 molecules per sodium alginate unit) [21]. Based on their results it may be estimated that in the external part of the matrix (*l* ~ 9 mm), the polymer and water formed a solution.

According to the theory of Peirce et al. and its extensions (Magne et al., Okubayashi et al.) concerning the mechanism of water sorption by polysaccharides, it could be assumed that at low total water content, it was adsorbed on polymer chains through hydrogen bonds with the sorption centers (hydrophilic groups, sodium ions, etc.) [24,25,26]. When freezing water was observed (the matrix region for *l* above 1 mm), the places capable of binding nonfreezing water in the matrix were already saturated. The uptake of additional water molecules spread apart the sodium alginate chains and caused the exposition of new polymer sorption sites in the regions situated closer to the matrix surface, resulting in higher values of *WNF_w/w_* in those regions [20,27].

The KF and DSC data represented slices approximately one-millimeter thick. Higher resolution in the matrix volume region located at *l* = 0-4 mm was not feasible due to technical limitations of the device used for tablet hydration as well as destructive character of sample preparation. Moreover, the external part of the matrix protruding beyond the holder was treated as a source of one sample for KF and DSC analysis—it mainly contained a viscous “gel-like” substance unsuitable for slicing. However, it is worth noting that such a spatial characterization of a polymer matrix under hydration using KF and DSC was not presented previously. In MRI (MSME and UTE) analysis the whole matrix cross-section was easily visible. Nondestructive MRI methods allowed observing the changes of matrix properties through the tablet cross-section with an order of magnitude higher resolution (i.e., 0.1 mm).

**Figure 6:**
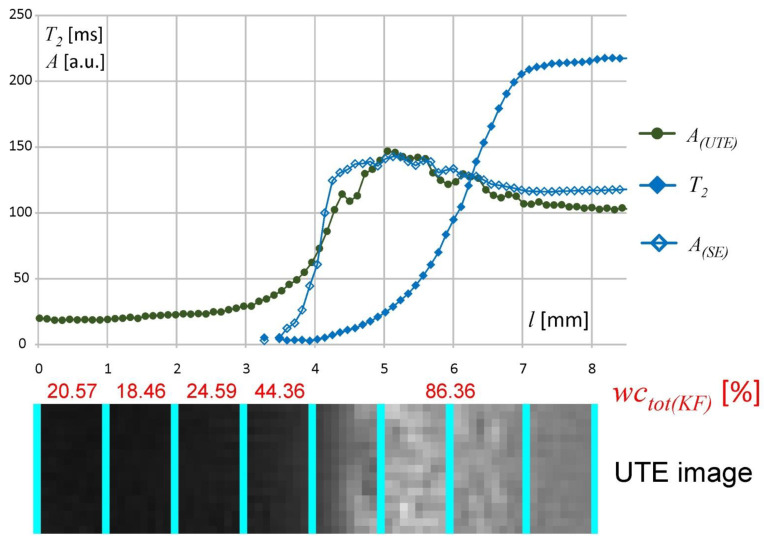
The juxtaposition of results obtained with MR imaging, (ultrashort echo time) UTE and multislice spin-echo (MSME) sequences together with KF results at 1 h of hydration (*A_(UTE)_*—UTE image intensity profile, *A_(SE)_* and *T*_2_—amplitude and effective *T*_2_ relaxation time profiles obtained from the multi-echo spin-echo experiment).

The UTE sequence application was proposed as a link between the KF, DSC and MSME results. Using UTE, the signal acquisition started a very short time after stimulation, i.e., 300 µs. This allowed protons to be imaged in regions with very short *T*_2_ relaxation times in the least hydrated regions of the matrix (including mobilized polymer and bound water). Therefore, it was possible to acquire an MR signal from the inner part of the matrix starting from *l* = 0 mm. UTE and multi-echo spin-echo (MSME) signal intensity profiles scaled in arbitrary units are juxtaposed in Figure 6 together with the KF results and the corresponding UTE image (results obtained at 1 h of hydration). The UTE and MSME signal intensity profiles match each other in the *l* = 4–7 mm spatial range. The MSME sequence allowed signal acquisition at 3.5 ms and only in freezing water (DSC based terminology), i.e., only higher mobility protons could be detected starting from *l* ≈ 3 mm. Protons with less mobility were not detected in the MSME sequence due to detection limitations, but multi-echo spin-echo (MSME) data complemented KF and DSC data in the highly hydrated regions of the matrix. The signal intensity in the spatial range containing hydration medium (water) (*l* > 7) differed between the UTE and multi-echo spin-echo (MSME) profiles due to different *T*_1_ weighting. The apparently higher water content in the hydrated matrix than in the bulk solvent (water) is also a result of *T*_1_ weighting (i.e., the bulk solvent was slightly attenuated). Despite detection limits (i.e., TE = 3.5 ms) the quantitative measurement of the effective *T*_2_ relaxation and corresponding signal amplitude was performed and gave a link between water content and its molecular mobility. Using multi-echo spin-echo imaging one pool (nonfreezing) of water was found at single pixel level, characterized by signal amplitude (*A*) and corresponding effective *T*_2_ relaxation time. Both values decreased towards *l* = 0 and *T*_2_ had the highest values *T*_2_ > 200 ms in the matrix regions adjacent to the bulk solvent (*T*_2_ ≈ 250). 

**Figure 7:**
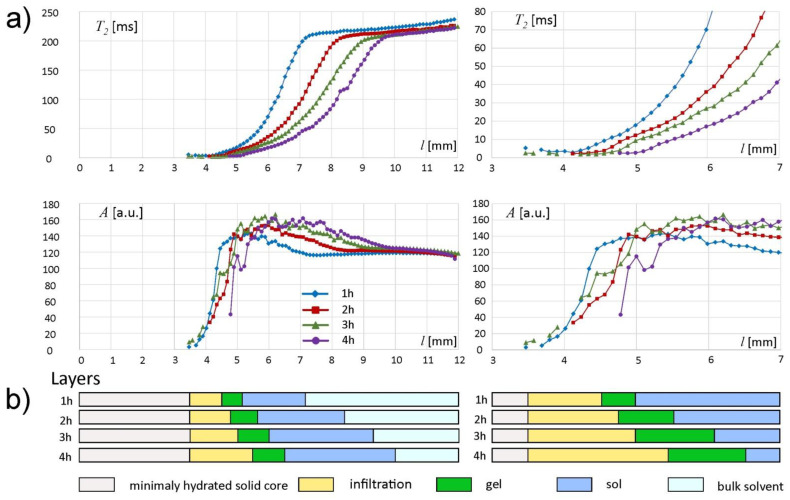
Results of MR multi-echo spin-echo imaging: (**a**) signal amplitude (*A*) and *T*_2_ relaxation time profiles in full spatial range (**left**) and restricted to *l* = 3–7 mm (**right**); (**b**) interpretation of the results in terms of layers.

The complete results and interpretation of multi-echo spin-echo (MSME) imaging are presented in Figure 7. The whole matrix spatial range was separated into layers (important note: “slices” refer to KF/DSC sampling, while “layers” denote interpretation results based on multi-echo spin-echo MRI). The deepest part of the matrix, i.e., for *l* < 3 mm, gave no signal when using MSME sequence. This region was denoted as minimally hydrated solid core (Figure 7b). According to KF results—starting from 1 h of incubation, the hydration level of the deepest regions of the matrix was higher than the initial one (i.e., as assessed for the unhydrated matrix). Two pools of water were present in this layer. The first, present in unhydrated matrix, was connected with polymer binding sites in its hydration shell and was assigned as nonfreezing according to the DSC results. The second pool was water absorbed by the matrix during hydration. According to the DSC results this second pool of water contributed both to nonfreezing and to freezing water and it preceded a steep hydration front. Starting from the spin-echo signal detection limit at *l* ≈ 3 mm, the amplitude increased steeply towards the external border of the matrix—in this region *T*_2_ < 10 ms (mostly set at the lower limit). This layer was denoted as an infiltration layer and was marked yellow in Figure 7. The approximate width of the layer was 1, 1.25, 1.5 and 1.75 mm at 1, 2, 3 and 4 h of incubation, respectively. Despite high water content, its molecular mobility was still very low in terms of effective *T*_2_ relaxation time. The water content reached almost maximum at corresponding *T*_2_ ≈ 10 ms. These spatial locations can be assigned as a second (right) border of infiltration layer, very similar to those observed for HPMC-based matrices [28,29] and can be regarded as the beginning of the fully hydrated part of the matrix. The position of this front was 4.5 mm at 1 h, 4.75 mm at 2 h, 5.0 mm at 3 h and 5.25 mm at 4 h of hydration (Figure 7a). The infiltration layer tended to be more gradual at consecutive hydration times. The fully hydrated part of the matrix could be further separated into two distinct layers. From this spatial position the *T*_2_ of the detected water increased linearly. It should be noted that generally, increase in *T*_2_ time means an increase in proton mobility. The layer characterized by linear spatial increase in *T*_2_ was denoted as gel and marked green in Figure 7. The approximate width of the layer was 0.5, 0.75, 1 and 1.25 mm at 1, 2, 3 and 4 h of incubation, respectively. The position of switch point in spatial *T*_2_ increase (the change from linear to nonlinear) was approximately 5.0 mm at 1 h, 5.5 mm at 2 h, 6.0 mm at 3 h and 6.5 mm at 4 h of hydration. The layer characterized by nonlinear spatial *T*_2_ increase was marked blue. It can be assumed that switch-point position marks the gel/sol transition. Therefore, the layer was denoted as sol. The approximate width of the layer was 2.25, 2.75, 3.25 and 3.25 mm at 1, 2, 3 and 4 h of incubation, respectively.

The beginning of the bulk solvent is marked by another switch-point, i.e., the blurry border between concentrated sol and bulk solvent— the beginning of plateaus both in amplitude and *T*_2_ profiles could be identified (Figure 7a). The *T*_2_ plateau started at a value of about 210 ms for each hydration time. The plateau started at *l* = 7.25 mm at 1 h of hydration, *l* = 8.25 at 2 h, 9.25 at 3 h and *l* = 9.75 at 4 h of hydration. The bulk solvent is marked light blue in Figure 7b. The *T*_2_ plateau rose slightly towards higher values of *l.* It was related to a decrease in the content of dissolved polymer particles in bulk solvent (higher *T*_2_ values means less polymer dissolved).

The maximum values of amplitude *A* of the spin echo envelope signals in the matrix region adjacent to the hydration medium increased from approximately 143 *a.u.* for *l* = 7.25 mm after the first hour to approximately 166 *a.u.* for *l* = 8.2 mm after the fourth hour of the experiment. This indicates that new water molecules penetrated the gel/sol regions of the matrix, increasing water content in these regions.

The extensive results presented above can be summarized to give a compact overview of the alginate matrix under unilateral hydration. The hydration in the minimally hydrated layer preceded the steep hydration front. The minimally hydrated layer gained water starting from the first hour of incubation (5–10% *w*/*w*) and at 4 h the total water content was 29–39% with a nonfreezing pool of 28–29% as assessed by KF and DSC. The infiltration layer with the steep hydration front was characterized by low water mobility in terms of effective *T*_2_ relaxation times (*T*_2_ < 10 ms). The water content at the beginning of the infiltration layer (*l* = 3–4 mm) was 45–47% (1–3 h of hydration) and at 4 h it reached about 50% with nonfreezing pool water molecules of 28%. The water content reached almost maximum at *T*_2_ circa 10 ms. Despite full hydration, the first part of the fully hydrated layer (gel) was characterized roughly by *T*_2_ < 20 ms (with linear spatial increase towards external part of the matrix), which reflected low water mobility. The second part of the fully hydrated layer with *T*_2_ between 20 and 200 ms (with nonlinear spatial increase) was denoted as sol. The water content at near the sol/bulk solvent interface was 85–86% from the first hour of hydration with a nonfreezing water pool of 11% at 4 h as assessed using KF and DSC.

The sodium alginate matrix tablet could not be regarded as a medicine itself because it did not contain any active substances. However, its analysis showed the complexity of the “picture” formed during hydration. It gives a good starting point for further studies of more complex systems (e.g., oral controlled release drug delivery systems), where the drugs and additional excipients could introduce additional factors modifying the water transport and interaction with the matrix.

## 4. Conclusions

The spatiotemporal characterization of alginate matrix tablets under unilateral hydration using hybrid, destructive (KF, DSC) and nondestructive (MRI), analysis was proposed. Water distribution and interaction with the polymer matrix was performed on nonequilibrated samples (mainly for low hydration regions) using Karl Fischer and DSC with rough spatial resolution (1 mm slices). Multi-echo spin-echo magnetic resonance imaging with 10-fold higher resolution was performed in a nondestructive manner for *T*_2_ relaxation time mapping (mainly for high hydration regions).

The analysis of the hydration behavior of the alginate matrix showed initial quick water penetration to the deeper layers of the matrix combined with the close interaction with the polymer—presence of nonfreezing water. The increase in the water content in the particular slices was gradual and when it exceeded 28–29%, the freezing water pool was built. The presence of freezing water was observed even in the external slices of the matrix tablets; however, its content was lower. Using MRI it was possible to separate the matrix regions (layers) of distinct spatial trends in signal parameters (amplitudes and *T*_2_ relaxation times). The minimally hydrated layer, infiltration layer and fully hydrated matrix layer with two sublayers (i.e., gel and sol) were identified based on T_2_ mapping.

The developed hybrid experimental methodology allowed combining physicochemical information from particular regions of the hydrated matrix system with molecular information characterizing proton mobility and its distribution. It allowed detection of hydration-related features characteristic of the particular polymeric (in this case alginate) matrix and in consequence gave a deeper understanding of the hydration processes in the alginate matrix system. The analytical approach as used for the pure alginate compressed matrix may be easily used to characterize other pharmaceutical, controlled release systems based on hydrophilic polymers e.g., alginate matrices with the addition of substances of different solubility.

## Data Availability

No new data were created or analyzed in this study. Data sharing is not applicable to this article.
